# Diagnostic Accuracy of Early Computed Tomographic Angiography for Visualizing Medium Sized Inferior and Posterior Projecting Carotid System Aneurysms

**DOI:** 10.5812/kmp.iranjradiol.17351065.3135

**Published:** 2011-11-25

**Authors:** Hooshang Saberi, Mohammad Hashemi, Zohreh Habibi, Ali Tayebi Meybodi, Seyed Ali Fakhr Tabatabai, Hazhir Saberi, Sofia Saboori

**Affiliations:** 1 Department of Neurosurgery, Brain and Spinal Cord Injuries Repair Research Center, Imam Khomeini Hospital, Tehran University of Medical Sciences, Tehran, Iran; 2 Department of Radiology, Medical Imaging Center, Imam Khomeini Hospital, Tehran University of Medical Sciences, Tehran, Iran; 3 Tooska Medical Imaging Center, Tehran, Iran

**Keywords:** Angiography, Digital Subtraction, Helical CT, Intracranial Aneurysm

## Abstract

**Background:**

Conventional angiography, generally referred to as intra-arterial digital subtraction angiography, still remains the gold standard reference method for the diagnosis of intracranial aneurysms, helical computed tomography angiography (CTA) is a new non-invasive volumetric imaging method.

**Objectives:**

This study was conducted to screen patients presenting with subarachnoidhemorrhage by CTA before conventional digital subtraction angiography (DSA) and subsequently comparing the results for various aneurysm projections.

**Patients and Methods:**

In a prospective study, 99 consecutive patients with an initial diagnosis of subarachnoid hemorrhage were screened for aneurysms with CTA followed by conventional DSA. There were 17 cases with negative angiograms in whom repeat angiograms, three months later were negative for 15 cases, while two cases were found to bear aneurysm on the repeat examination. Eighty two patients had at least one proven aneurysm on initial DSA and two on the repeat angiogram. Out of 84 patients, five underwent endovascular treatment and 79 patients who underwent surgical clipping were considered for projection evaluation.

**Results:**

Sensitivity of CTA was 98.78% (95% confidence interval [CI], 93.4-99.7%), while the specificity was 100% (95% CI, 81.57-100%) and the kappa coefficient of agreement between CTA and DSA was 96.5%. The most significant discrepancies with DSA findings were for visualizing the projection of inferior and posterior projecting proximal anterior circulation aneurysms.

**Conclusions:**

Helical CTA was in good concordance with DSA for screening of cerebral aneurysms; however, for exact visualization of the aneurysm neck and its projection, especially if it is inferior or posterior, DSA remains the gold standard.

## 1. Background

Conventional angiography, generally referred to as intra-arterial digital subtraction angiography (DSA), still remains the gold standard reference method for the diagnosis of intracranial aneurysms [1][2]. DSA is associated with a false negative rate of approximately 5-10% [3]. This wide range may be attributed to the physical limitations of angiographic equipments, which could hardly produce optimal projections for some intracranial aneurysms [4][5]. Meanwhile an aneurysm has been discovered, visualization of the aneurysm neck and its connection to the parent artery has been reported as impossible in 14% of the cases [4], which definitely has an untoward impact on the diagnosis and management of the patients [6]. On the other hand, DSA as the screening method is more invasive and deserves highly experienced specialists. Besides, the procedure requires arterial puncture and manipulation. The risk of arterial puncture and catheter manipulation is low, but significant (1%). Besides, the incidence of new permanent neurological deficit is about 0.5% [7].

Helical computed tomography angiography (CTA) is a new non-invasive volumetric imaging method. In this method, obviating the need for arterial puncture and/or catheter manipulation, the images are three dimensionally reconstructed, without a significant risk for the patient [8]. Performing this procedure once, the data may be visualized from indefinite perspectives in two dimensional and three dimensional modes, simplifying aneurysm detection [9][10]. Some studies have shown that this method may visualize the aneurysm even when DSA has had false negative results [11]. Nevertheless, DSA remains the gold standard method for evaluation of cerebral arteries. Recent studies show that employing 3D-CTA has had privileges in screening for cerebrovascular disorders [12], paving the way for three-dimensional study of the arterial tree [13].

Although recently the image quality of 3D-CTA is rapidly improving, DSA still remains the gold standard test for the diagnosis of intracranial aneurysms and planning of treatment. Employing 3D-CTA for careful diagnosis of cerebral aneurysms and concordance with intra-operative findings has been of utmost importance for neurological surgeons [14]. On the other hand other studies have shown that CTA has limitations in precision for defining the neck projection and arterial branching pattern [15][16]. Noteworthy in the recent 5 years, with rapid software developments in three dimensional visualization, intracranial aneurysms have been more carefully defined [17]. Employing 3D-CTA could produce reliable imaging for diagnosis of intracranial aneurysms and planning noninvasive therapeutic interventions [6].

## 2. Objectives

The aim of this study was to survey the diagnostic accuracy of helical 4-row detector CTA with first conventional DSA for cerebral aneurysms. Meanwhile we have assessed the accuracy of CTA and DSA with intra-operative results for aneurysm neck projection.

## 3. Patients and Methods

### 3.1. Patients

In a prospective study, with the medical ethics committee certificate, from 2005 to 2007, amongst patients presenting with subarachnoid hemorrhage into the emergency department, 99 consecutive patients with the initial diagnosis of subarachnoid hemorrhage were enrolled into the study and screened for aneurysms with CTA followed by conventional DSA who were considered for diagnostic accuracy of CTA in comparison with the first DSA for the detection of aneurysm. There were 17 cases with negative first angiograms; among these patients the repeat angiograms three months later were negative for 15 cases, while two cases were found to bear aneurysm on repeat examination; therefore, 84 patients had at least one aneurysm to be treated. Out of 84 patients, five patients each harboring one aneurysm underwent endovascular treatment and 79 patients who underwent surgical clipping were considered for projection study. In 70 patients, aneurysms were single (88.61%), in seven patients (8.86%) there were two aneurysms and in two patients (2.53%) there were three aneurysms; thus, we had totally 90 aneurysms among them. The cerebral arteries were evaluated for aneurysms with both DSA and CTA by two independent neuroradiologists for those having informed consent for the study. Patients without an informed consent and those accomplishing only one of the studies and patients in an emergency situation and/or medical contraindication for high dose iodine administration were excluded. In addition, patients having documented coagulopathy were excluded from the study.

### 3.2. Techniques

**Figure 1:**
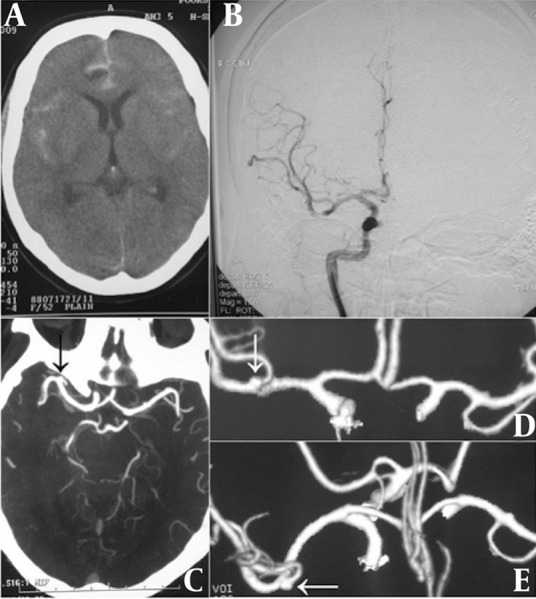
A 45-Year-Old Man With Sudden Onset Headache and Drowsiness. A, Computed tomography demonstrates subarachnoid hemorrhage. B, Digital subtraction angiogram detects anomalous angioarchitecture and vasospasm of post-trifurcation MCA. C, CT angiogram reveals a shadow in MCA trifurcation (black arrow) resembling sacular aneurysm. D and E, More evident in 3D reconstructions (white arrows)

Cerebral arteries were examined on DSA and CTA suits simultaneously. DSA study was performed with Innova 4100 flat panel system. An anesthesiologist visited all the cases and sedation was performed if necessary. Trans-femoral catheterization of both common carotid and bilateral vertebral arteries was performed. Ultravist 300 was employed as the contrast agent. Images were obtained from arterial to the venous phase and a maximum of 9 mL of contrast was used for each view. Anteroposterior, oblique, lateral and rotational images were obtained if necessary (Figure 1).

CTA was obtained with GE 2004 light speed QXI 4-row D system. Initially an axial brain CT was obtained as baseline information. Then 100 mL of nonionic contrast (Visipaque or Ultravist) was administered through a gauge 20 intravenous line with the speed of 5 mL/s. The scanning started with the bolus triggering technique at the level of the aortic arch. Axial slices were taken with 1.25 mm thickness and overlapping of 0.625 mm. The reconstruction was performed in the DW4.1 workstation.

### 3.3. Evaluations

The obtained images were reported by two independent neuroradiologists. In the presence of documented studies the patients were scheduled for clipping. During cerebral arterial dissection, the number, location and projection of the aneurysms were examined and documented by the operating neurosurgeon. Finally, the diagnostic accuracy of CTA for determination of the number, location and projection of the aneurysms were compared with DSA and intra-operative findings as the gold standard. Based on ethical issues, we were not allowed to explore all cerebral arteries; therefore, in this study we were able to only scrutinize aneurysms depicted by DSA.

### 3.4. Statistics

Sensitivity, specificity and accuracy rates were determined. Statistical analysis was performed with chi square or Fisher’s exact test and a P < 0.05 was assumed to be significant.

## 4. Results

The mean age at presentation was 49.06 ± 13.6 (range, 20-85 years) and 50.63% of the patients were male. The mean aneurysm size (largest diameter regardless of aspect ratio) was 6.30 ± 1.26 millimeters on DSA, 6.26 ± 1.23 on CTA, and 6.34 ± 1.22 intra-operatively, demonstrating no statistically significant differences. Among 82 patients with positive first DSA, one was missed in CTA and none of the 17 negative cases of first DSA were positive in CTA. The diagnostic accuracy indices including sensitivity, specificity, positive predictive value and negative predictive value were 98.1%, 91.3%, 92.8% and 97.7%, respectively. We also calculated the kappa agreement coefficient (Table 1).

**Table 1:** Diagnostic Accuracy Indices of CTA Versus DSA and the Relevant Confidence Interval

	**No.**	**95% CI a**
Sensitivity	0.988	0.934-0.998
Specificity	1	0.816-1
PV a		
Positive	1	0.955-1
Negative	0.944	0.742-0.99
LR a		
Positive	-	-
Negative	0.012	0.002-0.086
Accuracy	0.989	0.945-0.998
Kappa	0.965	0.898-1.03

^a^ Abbreviations: CI, Confidence interval; LR, Likelihood ratio; PV, Predictive value

The data regarding the prediction of aneurysm projection by CTA method has been tabulated in Table 2. As seen, the highest non-concordance rate between CTA and DSA as well as intra-operative findings in terms of projection was on the inferior projection followed by the posterior projection. Those with negative results were re-evaluated with DSA two months later. There were no aneurysms detectable on re-evaluation. These cases were either uncontrolled hypertensives, or those who had perimesencephalic subarachnoid blood distribution. In our patients, there was no case of discrepancy between DSA and intraoperative findings in terms of aneurysm projection.

**Table 2:** Relative Comparison of the Frequency of Various Aneurysm Projections Observed on Computed Angiography and Digital Subtraction Angiography

	**Relative Frequency in DSA a, %**	**Relative Frequency in CTA a, %**	**Relative Frequency Intra-Operatively, %**	**Non-Concordance Between CTA and Intra-Operative Findings, %**
Anterior	41.11	43.33	35.56	17.95
Posterior	5.56	6.67	4.44	33.33
Superior	22.22	23.33	21.11	9.52
Inferior	22.22	21.11	12.22	42.11
Lateral	61.11	61.11	46.67	23.64

^a^ Abbreviations: CTA, Computed tomography angiography; DSA, Digital subtraction angiography

## 5. Discussion

According to the results of the present study, there was single aneurysm in 70 (88.61%) patients, double aneurysms in seven (8.86%) patients and triple aneurysms in only three (2.53%) patients. Feng et al. [14] in a similar study of 24 patients, found single aneurysms in 21, double aneurysms in two and triple aneurysms in one patient. They also reported a round shape in 20, ellipsoid in five and lobulated in three patients, having a good correlation between CTA report and operative findings. The same correlation was also observed in our patients. The mean aneurysm size on DSA and CTA revealed no statistically significant difference (6.30 ± 1.26 vs. 6.26 ± 1.23).

According to Feng et al. [14], the aneurysm size (5.9 mm) was closely correlated to intra-operative measurements (P < 0.001). In the comparison between DSA, CTA and magnetic resonance angiography, the 3D CTA depicted aneurysms and parent vessel anatomical relationships more accurately. Our findings yielded the mean aneurysm neck size of 3.55 ± 1.06 mm on DSA, while the result was 3.51 ± 1.08 mm on CTA showing no statistically significant difference. Preoperative determination of the aneurysm neck size is highly important for surgical decisions. However, some studies revealed inter-observer variability for the estimation of aneurysm neck size by CTA [18].

The study by Feng et al. [14] revealed a smaller aneurysm neck size on CTA compared to intra operative actual findings; however, the difference was not significant. The phenomenon may be explained, because CTA actually shows the inner diameter of the aneurysm neck. In all our 79 patients with medium sized aneurysms, DSA and CTA successfully discovered all aneurysms correctly and the findings were verifiable by operative findings. Considering any examination as an adjunct for DSA in the diagnosis of asymptomatic aneurysms mandates very high sensitivity, because a neglected aneurysm is a potentially dangerous situation, threatening the patient’s life. Besides, for patients who do not prefer to undergo DSA and/or surgery, a very high specificity rate is also required, especially for diagnosis and determination of very small intra-cranial aneurysms. According to the report by Anderson et al. [19] the sensitivity and specificity of CTA for pre-operative diagnosis of ruptured and un-ruptured aneurysms were 84% and 100%, respectively.

In the study by Karamessini et al. [20], they reported a sensitivity of 88.7%, specificity of 100%, positive predictive value of 100%, negative predictive value of 80.7% and an accuracy rate of 92.3% for CTA studies. On the other hand for DSA studies, a sensitivity of 87.8%, specificity of 98%, positive predictive value of 97.7%, negative predictive value of 89.1% and an accuracy rate of 92.9% were calculated, provided aneurysms with a diameter of 3mm or more are considered. The sensitivity of CTA would be in the range of 93.3% to 100% which equals to that of DSA. In the study by Kangasniemi et al. [21], a sensitivity of 96% and a specificity of 97% were reported for CTA regarding aneurysm detection.

Chappell et al. in their meta-analysis [22] of 21 studies about CTA on 1251 patients, reported a 93.03% (range, 75.4%-100%) sensitivity and a 87.8% (range, 0-%100) specificity. Stratifying the studies on the basis of the number of patients, the mean sensitivity decreased to 92.7% and the specificity decreased to 77.2%, so they reported DSA as the standard method; however, CTA has less risk and discomfort for the patient, but its statistics profile is far below that of DSA. Potentially, the combination of bone subtraction (by means of double scanning or dual-source multidetector CT) and increasing the number of detectors for multidetector CT angiography could lead to the diagnostic accuracy that is equivalent to the combination of DSA and 3D rotational angiography [23]. CTA has no risk for stroke. It is acquired within approximately 5 to 20 seconds. Furthermore, it uses less contrast than a full “4-vessel” angiogram. DSA has a sterile setup, requires arterial access and consists of selective catheter manipulations [24]. Compared with conventional CTA, subtraction CTA is a more powerful imaging method that seems to be equivalent to 2D-DSA for the detection and pre-treatment planning of intracranial aneurysms [24], although further studies may be necessary to establish the hypothesis. 3D-DSA has obviated the limitations in visualizing the aneurysm neck in various directions obtained from 2D-DSA, In addition it should be kept in mind that DSA methods are more time consuming and the results are less reliable in agitated patients. According to Chen et al., 16-slice CTA shows promising diagnostic accuracy that appears to be comparable with 2D-DSA for the detection of suspected intracranial aneurysms and 16-slice CTA is sensitive enough to replace 2D-DSA in detecting aneurysms [25]; however, the study does not notify anatomical clarification of the aneurysm. It should be kept in mind that aneurysm size may affect the obtained sensitivity and specificity rates. Van Gelder et al. [26] showed that the sensitivity of CTA may vary from 53% [95% confidence interval (CI), 44-62%] for 2 mm aneurysms to 95% (95% CI, 92%-97%) for 7 mm aneurysms, while the overall specificity was 98.9% (95% CI, 91.5%-99.99%). In another observation, Korogi et al. [15], showed a mean sensitivity of 64% for very small intracranial aneurysms and 100% for large aneurysms for CTA reported by four readers in the survey of 47 aneurysms.

Yusal et al. concluded that 16-row multislice CTA is equally as sensitive as DSA in the detection of intracranial aneurysms greater than 3 mm. Multidetector CTA has also high sensitivity in detecting aneurysms less than 3 mm with the help of technical advances and increasing the reader’s experience [27][28]. Recently, 64-multi slice 3D-CTA was shown to have an equivocal diagnostic advantage to DSA, with the specificity and positive predictive value (PPV) being 100%, which is far beyond our results, while the significantly lower negative predictive value implied that CTA may not be a suitable method to rule out the vascular ectasia [29]. Again visualization anatomical details should be considered.

According to some studies, CTA has had high sensitivity and specificity for residual aneurysm detection so CTA has been useful for the follow-up of clipped aneurysms [30]. This may pave the way for future studies for follow-up of clipped aneurysms. When DSA and CTA are compared in the first 3 days, 16-row multiline CTA has been found to have a sensitivity, specificity and accuracy of 100% for patients harboring intracranial aneurysms smaller than 5mm [31]. This may be explained because vasospasm has not yet taken place. Conventional CTA has been reported to have a limited sensitivity in detecting tiny aneurysms and those adjacent to bony structures; on the other hand, subtraction CTA may obviate these problems, yielding results similar to DSA as was the case in our patients who had 3D subtraction images to delete the bone signal from the images [24][32]. In our study, the kappa coefficient was found to be 0.9 for aneurysms larger than 5 mm. Meanwhile in the Lubicz et al. series, CTA was shown to have a kappa coefficient of 0.753 with acceptable sensitivity and specificity; however, the rates are lower for small intra-cranial aneurysms [33]. Therefore, CTA has been accurate for aneurysms larger than 4 mm in size if performed within hours after SAH, although it may miss small aneurysms [23].

Finally, our results disclosed the sensitivity, specificity, and accuracy rates of CTA to be acceptable as a screening procedure to find out the presence of intracranial aneurysms. Taking into consideration the non-invasiveness and the three dimensional view, we may recommend CTA as a feasible screening method according to the clinical judgment of the responsible physician. Nonetheless, in aneurysms smaller than 5 mm and those embedded in the skull base, the true non-concordance rate between CTA and DSA may be higher. The obtained values for CTA may be improved employing more detectors and dual source machines combined with subtraction technology.
